# PA28α overexpressing female mice maintain exploratory behavior and capacity to prevent protein aggregation in hippocampus as they age

**DOI:** 10.1111/acel.13336

**Published:** 2021-03-15

**Authors:** Julia Adelöf, John Wiseman, Madeleine Zetterberg, Malin Hernebring

**Affiliations:** ^1^ Department of Clinical Neuroscience Institute of Neuroscience and Physiology Sahlgrenska Academy at the University of Gothenburg Gothenburg Sweden; ^2^ Discovery Biology, Discovery Sciences BioPharmaceuticals R&D AstraZeneca Gothenburg Sweden

**Keywords:** aging, exploratory behavior, F2 hybrid transgenic mice, healthy aging, learning and memory, PA28αβ, proteasome activator, protein aggregation

## Abstract

With age, protein damage accumulates and increases the risk of age‐related diseases. The proteasome activator PA28αβ is involved in protein damage clearance during early embryogenesis and has demonstrated protective effects against proteinopathy. We have recently discovered that adult female mice overexpressing PA28α (PA28αOE) have enhanced learning and memory, and protein extracts from their hippocampi prevent aggregation more efficiently than wild type. In this study, we investigated the effect of overexpressing PA28α on aging using C57BL/6N×BALB/c F2 hybrid mice. We found that the hippocampal anti‐aggregation effect was maintained in young adult (7 months) to middle‐aged (15 months) and old (22 months) PA28αOE females. While the PA28αOE influence on learning and memory gradually decreased with aging, old PA28αOE females did not display the typical drop in explorative behavior—a behavioral hallmark of aging—but were as explorative as young mice. PA28αOE lowered PA28‐dependent proteasome capacity in both heart and hippocampus, and there was no indication of lower protein damage load in PA28αOE. The life span of PA28αOE was also similar to wild type. In both wild type and PA28αOE, PA28‐dependent proteasome capacity increased with aging in the heart, while 26S and 20S proteasome capacities were unchanged in the timepoints analyzed. Thus, PA28αOE females exhibit improved hippocampal ability to prevent aggregation throughout life and enhanced cognitive capabilities with different behavioral outcomes dependent on age; improved memory at early age and a youth‐like exploration at old age. The cognitive effects of PA28αβ combined with its anti‐aggregation molecular effect highlight the therapeutical potential of PA28αβ in combating proteinopathies.

## Graphical abstract

PA28αβ has been linked to diverse physiological functions and its molecular role can be separated into a 20S proteasome stimulating (Function A) and a chaperone‐like (Function B) activity. PA28αOE female hippocampi exhibit increased anti‐aggregation activity throughout the life span (Function B). Behaviorally, these mice have improved memory at young age and the healthy aging trait of enhanced exploration at old age.

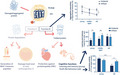

## 1 INTRODUCTION

As we age, damage to proteins and other macromolecules accumulates and causes deterioration of cellular and organ functions. Means to ensure a healthy proteome through maintained proteostasis is envisioned to prevent age‐related disease and to prolong life span (Basisty et al., 2018; Kennedy et al., 2014; Koga et al., 2011; Lopez‐Otin et al., 2013). PA28αβ is a protein complex with interesting proteostatic effects in this context. During early embryogenesis in mice, cells rid themselves of aging‐related deleterious protein damage (Hernebring et al., 2006), and this process requires PA28αβ (Hernebring et al., 2013). PA28αβ is important for recovery from re‐oxygenation injury in the mouse heart (Li, Horak, et al., 2011), and its overexpression increases viability upon oxidative stress of both neonatal rat cardiomyocytes (Li, Powell, et al., 2011) and mouse embryonic fibroblasts (MEFs; Pickering & Davies, 2012). PA28α overexpression also prolongs life span of mice suffering from a heart‐specific desmin proteinopathy (Li, Horak, et al., 2011), delays disease progression in two photoreceptor degeneration mouse models (Lobanova et al., 2018), and PA28αβ has been shown to increase proteasomal degradation of oxidatively damaged hemoglobin (Pickering & Davies, 2012).

In a phenotypic screen of mice overexpressing PA28α (PA28αOE), we found that PA28αOE females exhibit improved learning and memory (Adelöf et al., 2018). Multiple parameters demonstrated this effect, including both learning in the shuttle box passive avoidance test and intersessional habituation in an open‐field test. These improved cognitive functions correlated to a decreased depressive‐like behavior, as PA28αOE female mice also displayed a marked increase in mean active time in the forced swim test (Adelöf et al., 2018).

In this work, we investigate the effects on aging of PA28α overexpression, using the C57BL/6N×BALB/c F2 hybrid background (herein abbreviated F2 hybrid). Hybrid mice are preferable to use in aging studies, because (i) compared to inbreds, they are less prone to develop strain‐specific diseases at old age, (ii) they do not display strain‐specific behavior as inbreds, and (iii) the heterozygosity of F2 hybrids reflects heterogenetic populations better than inbreds (Adelöf et al., 2019; Miller & Nadon, 2000). The timepoints used to analyze progression of biochemical and behavioral changes are 7 months of age, which represent fully grown mature adults, 15‐month‐olds that are considered middle‐aged, and 22 months of age that constitute the old cohort (Adelöf et al., 2019; Flurkey et al., 2007). PA28α overexpression has been shown to stabilize PA28β at the protein level (Li, Powell, et al., 2011), and this applies to all PA28αOE tissues successfully examined for PA28β protein content: MEFs, striatum/frontal cortex (Adelöf et al., 2018), and eye lens (Hernebring et al., 2021).

PA28αβ is involved in proteasome‐dependent degradation, binding to and activating the proteolytic 20S proteasome core. However, the PA28αβ‐dependent 20S proteasome activity was not induced in PA28αOE mice. Instead, protein extracts of hippocampus from female PA28αOE mice exhibited enhanced ability to prevent aggregation (Adelöf et al., 2018), indicating a chaperone‐like function of PA28αβ in hippocampus, which previously has been found also in reticulocyte lysates (Minami et al., 2000). Hippocampus is central for memory formation and spatial navigation, and age‐related hippocampal degeneration is generally considered an important cause of cognitive decline at advanced age. We were therefore particularly interested in whether the reduced protein aggregation in hippocampus is maintained in PA28αOE females as they age; and if so, which effects this would have on markers of aging, life span, and health span.

## 2 RESULTS

### 2.1 Enhanced aggregation prevention in female PA28αOE hippocampal extracts is maintained with aging

**FIGURE 1:**
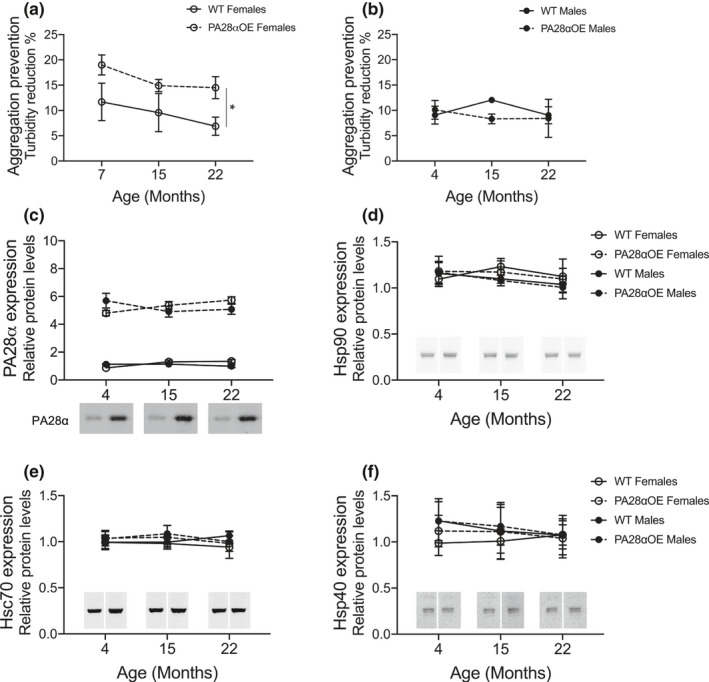
Aggregation prevention capacity of hippocampal protein extracts from (a) female mice at 7, 15 and 22 months of age and (b) male mice at 4, 15 and 22 months of age (all from F2 hybrid lifespan analysis except male 4‐month‐olds which were C57BL/6N). Turbidity reduction is a measure of how much the presence of hippocampal protein extracts from wild type (WT) and PA28αOE can prevent aggregation of heat‐sensitive luciferase at 42°C (*p = 0.0187; Mixed‐effects model). Values are mean ± SEM; *n* = 3–4. Protein levels of (c) PA28α, (d) Hsp90, (e) Hsc70, and (f) Hsp40 in hippocampal protein extracts from 4, 15, and 22 months old PA28αOE and wild type mice (all from F2 hybrid lifespan analysis except 4‐month‐olds which were C57BL/6N). Values are mean ± SD; *n* = 4 except for Hsp40 n_WTF4_ = 3. Insets are representative western blots of the different ages (cropped from the same membrane for each analysis)

As outlined in the Introduction, we previously found that protein extracts of hippocampus from 7‐month‐old (mature adult) female PA28αOE mice exhibit an enhanced ability to prevent aggregation compared to wild type (Adelöf et al., 2018). Littermates to these mice were followed in a lifespan study, and cohorts were analyzed in the same way as the 7‐month‐olds, at the ages of 15 months (middle‐aged) and 22 months (old). We found that the increased capacity of aggregation prevention in hippocampal extracts from PA28αOE female mice was maintained with aging (*p* = 0.0187, Mixed‐effects model; Figure 1a). In male mice, we observed no difference between wild type and PA28αOE (ns, Mixed‐effects model; Figure 1b).

### 2.2 The improved aggregation prevention of female PA28αOE hippocampus is not due to increased levels of Hsp90, Hsc70, Hsp40, or altered serum β‐estradiol

Aggregation of heat‐denatured luciferase is blocked in vitro by Hsp90 binding, and—in addition to Hsp90—the subsequent refolding requires Hsc70, Hsp40, and PA28αβ (Minami et al., 2000 and references therein). PA28αβ is implicated to provide a transitory binding site during transfer of the substrate from Hsp90 to Hsc70 in the early events of refolding (Minami et al., 2000). An upregulation of Hsp90, Hsc70, or Hsp40 in PA28αOE female hippocampi could explain the improved prevention of luciferase aggregation, and thus, we analyzed the levels of these proteins in hippocampal extracts.

While the amount of PA28α protein was increased fivefold in the hippocampus of PA28αOE mice (Figure 1c), there was no difference with genotype or age in protein levels of Hsp90 (Figure 1d), Hsc70 (Figure 1e), or Hsp40 (Figure 1f). These results identify PA28αβ as a limiting factor in handling heat‐inactivated luciferase in female—but not male—hippocampi. The sex discrepancy of PA28αOE proteostatic effect points toward a possible involvement of sex hormones. In addition, estrogens are known to impact hippocampal functions (Liu et al., 2008), and thus, we wanted to analyze whether PA28αOE females had altered estrogen signaling. However, we found that serum β‐estradiol levels and S105 phosphorylated estradiol receptor β levels did not differ between PA28αOE and wild type females in any of the cohorts (Figure S1), indicating that the chaperone‐like effect observed in PA28αOE female hippocampi is likely not linked to estradiol signaling.

### 2.3 Heart and hippocampus of PA28αOE mice exhibit decreased PA28‐20S activity

Because of the protective effects of PA28α overexpression upon cardiomyopathy and re‐oxygenation stress of the heart (Li, Horak, et al., 2011; Li, Powell, et al., 2011), we focused on heart, as well as hippocampus, for the further investigation of biochemical effects of PA28α overexpression during aging.

**FIGURE 2:**
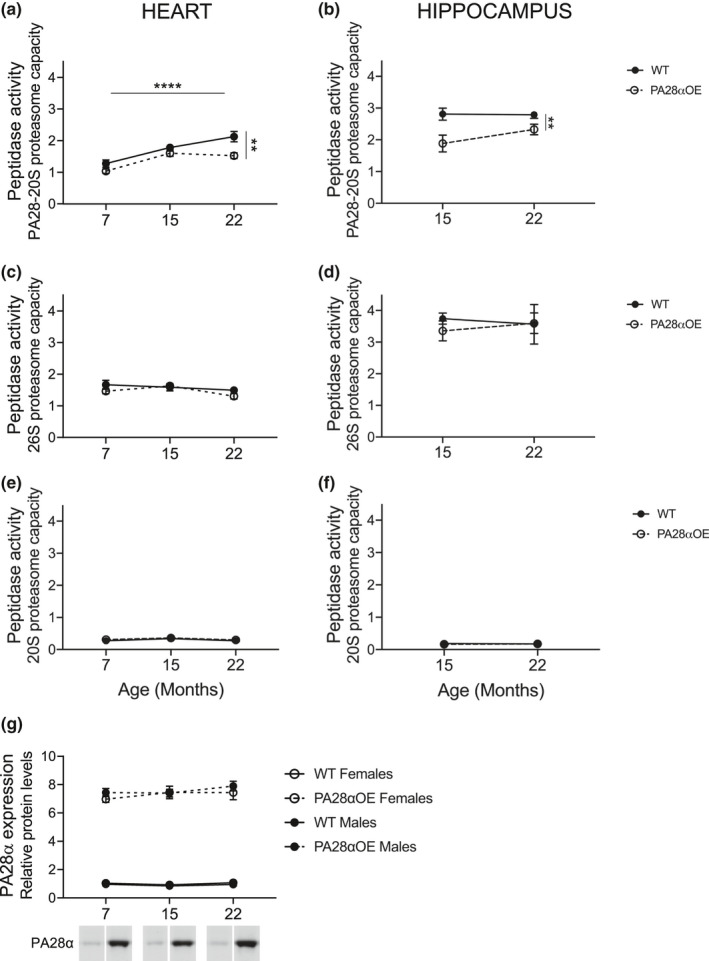
PA28‐20S proteasome capacity in (a) heart (****p_7–22_ < 0.0001, **p_WT‐OE_ = 0.0018; Mixed‐effects model) and (b) hippocampus (**p_WT‐OE_ = 0.0024; Mixed‐effects model), 26S proteasome capacity in (c) heart and (d) hippocampus, and 20S proteasome capacity in (e) heart and (f) hippocampus; all from F2 hybrid lifespan analysis. Values are mean ± SEM; heart: *n* = 11–12, hippocampus: *n* = 5–6. PA28α overexpression in (g) heart from PA28αOE and wild type (WT) mice from F2 hybrid lifespan analysis. Images below graph are representative western blots of protein extracts from the different ages (cropped from the same membrane)

The proteasome system consists of the 20S proteasome core that interacts with different activators regulating substrate access to 20S proteolytic chamber. The capacity of proteasome complex peptidase activity can be assayed using extraction and assay conditions that optimize the complex composition of interest (Hernebring, 2016). We unexpectedly found that PA28α overexpression decreased PA28‐20S proteasome capacity both in heart and hippocampus (p_heart_ = 0.0018, Figure 2a; p_hippocampus_ = 0.0024, Figure 2b; Mixed‐effects model), but had no effect on peptidase activities of 26S (Figure 2c,d) or 20S (Figure 2e,f) in either organ. During aging, PA28‐20S proteasome capacity increased in heart (*p* < 0.0001, Mixed‐effects model; Figure 2a) but not in hippocampus (Figure 2b), while 26S and 20S activity did not change with aging in either heart or hippocampus. Furthermore, on comparing heart PA28α protein levels in PA28αOE and wild type, we can conclude that PA28α was increased sevenfold in PA28αOE and that the age‐related induction of PA28‐20S proteasome capacity in heart was not caused by cumulative PA28α protein levels in this organ (Figure 2g).

### 2.4 PA28α overexpression does not reduce damage load in heart or hippocampus

PA28αβ has been repeatedly linked to protein damage control. Protein damage is expected to increase with aging and is generally considered at the core of age‐related organ deterioration (Anisimova et al., 2018; Koga et al., 2011; Levine, 2002). Thus, we assayed protein carbonylation, a common reporter of oxidized proteins, and the deleterious protein‐adduct metabolic byproduct advanced glycation end products (AGEs; specifically N^ɛ^‐carboxymethyllysine, CML) in the tissues of aging PA28αOE mice.

**FIGURE 3:**
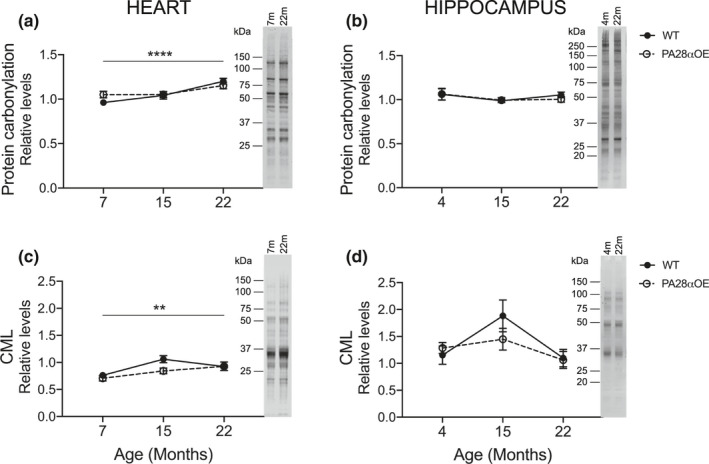
Protein damage levels in heart and hippocampus from young adult, middle‐aged and old wild type and PA28αOE mice. Carbonylated proteins in (a) heart (****p_7–22_ < 0.0001) and (b) hippocampus from PA28αOE and wild type (WT) mice. N^ɛ^‐carboxymethyllysine (CML) in (c) heart (**p_7–22_ = 0.003, Mixed‐effect model) and (d) hippocampus. All tissues were from the F2 hybrid lifespan analysis except 4‐month hippocampi that were from C57BL/6N. Values are mean ± SEM; heart: *n* = 11–12, hippocampus: *n* = 7–8. Images to the right of graphs are representative western blots of protein extracts from the youngest and oldest cohorts (size markers are approximations based on pre‐stained protein standards)

We found that protein damage increased with aging in heart (p_carbonyls_ = 0.0003; Figure 3a, p_CML_ < 0.0001; Figure 3c, Mixed‐effects model), but not in hippocampus, for either protein carbonylation (Figure 3b) or CML (Figure 3d). In addition, PA28α overexpression did not reduce protein damage load (Figure 3a,d), at least not in heart or hippocampus, which was unexpected considering its proposed role in degrading protein damage.

### 2.5 PA28α overexpression in PA28αOE does not prolong life span

**FIGURE 4:**
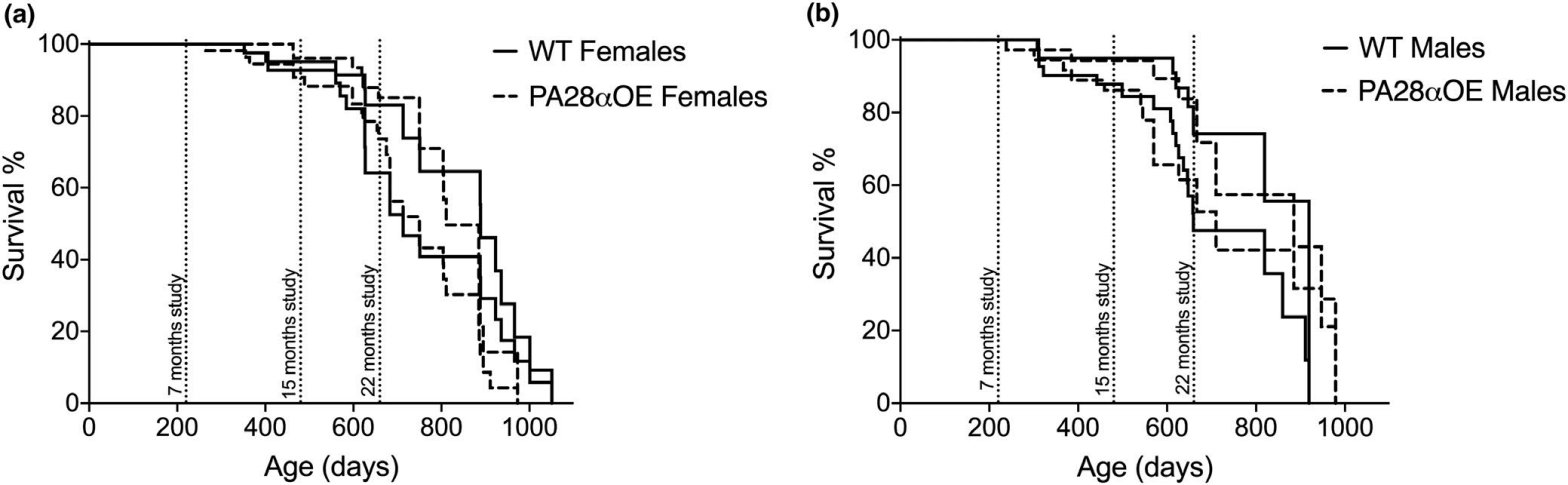
Lifespan analysis of PA28αOE and wild type C57BL/6N × BALB/c F2 hybrid mice displayed as interval of minimum and maximum survival curves. Lifespan of (a) female and (b) male PA28αOE mice (interval between dashed lines) and wild type mice (WT, interval between solid lines). Timepoints for behavioral analyses and organ harvesting of naïve cohorts of mature adult (7 months), middle‐aged (15 months), and old (22 months) mice are indicated. Females; n_WT_ = 51, n_OE_ = 60; males; n_WT_ = 48, n_OE_ = 41

While protein damage often correlates to life span (Anisimova et al., 2018), an ability of tissues to resist protein aggregation upon aging could possibly confer proteostatic effects benefitting longevity, even though the levels of protein damage is unaltered. However, there was no effect of PA28α overexpression in the PA28αOE mouse model on median or maximum life span, in either males or females (Figure 4; Table 1). Neither did analyses of body size, weight, body composition, and core temperature display any difference between wild type and PA28αOE (Table S1).

**TABLE 1:** Lifespan analysis of C57BL/6NxBALB/c F2 hybrid wild type and PA28αOE female and male mice to generate interval of natural life span in days, with variation denoting span between minimum and maximum survival curves (see Section 4)

	Mean life span	50% survival	7 months' survival	15 months' survival	22 months' survival
♀WT	803 ± 48	801 ± 88	100%	93 ± 2%	74 ± 10%
♀PA28αOE	774 ± 43	818 ± 68	100%	96 ± 2%	78 ± 8%
♂WT	760 ± 52	789 ± 130	100%	90 ± 5%	71 ± 11%
♂PA28αOE	765 ± 43	798 ± 88	100%	89 ± 5%	69 ± 10%

### 2.6 Enhanced memory and anti‐depressive‐like behavior in PA28αOE females are not maintained as they age

**FIGURE 5:**
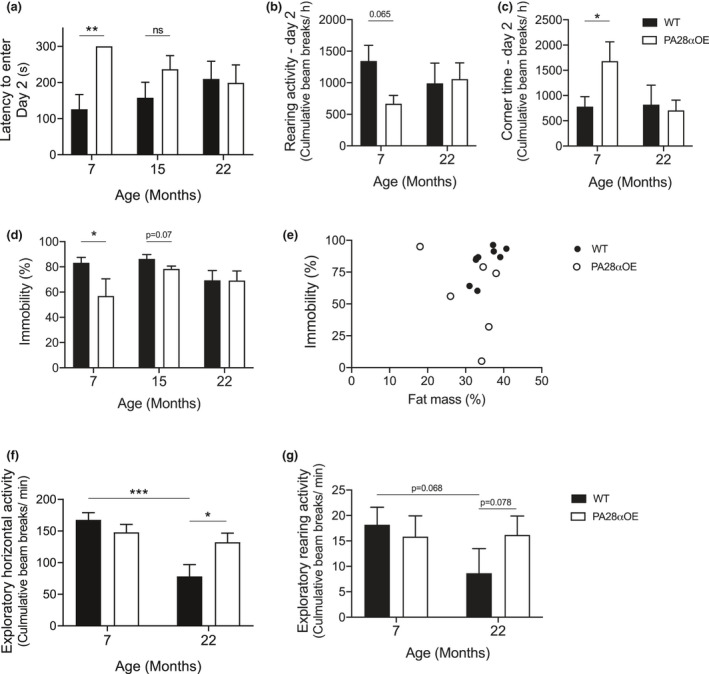
Behavioral testing of learning and memory, depressive‐like behavior and exploratory activity of wild type and PA28αOE female mice during aging (all from F2 hybrid lifespan analysis). (a) Learning and memory capacity as assessed by Shuttle‐box passive avoidance test, with 300 s being the maximum time of analysis (**p_WT7F‐OE7F_ = 0.0056; Mantel‐Cox). (b) Habituation analyzed on the day after naïve testing in the Activity box open‐field test by rearing activity (p_WT7F‐OE7F_ = 0.065; *F*(1, 14) = 3992), and (c) corner time (*p_WT7F‐OE7F_ = 0.034, *F*(1, 14) = 5498; two‐way ANOVA repeated measurements followed by Sidak test). (d) The forced swim test in which immobility is a measurement of depressive‐like behavior (*p_WT7F‐OE7F_ = 0.048 and p_WT15F‐OE15F_ = 0.07; Student's *t* test). (e) Correlation analysis of immobility to percent fat mass in the 7‐month cohorts (to control that forced swim results is not caused by differences in body composition). (f) Horizontal activity (***p_WT7F‐F22_ = 0.0006; *p_WT22F‐OE22F_ = 0.036; Student's *t* test), and (g) rearing activity (p_WT7F‐22F_ = 0.068; p_WT22F‐OE22F_ = 0.078; Mann–Whitney) during the first 5 naïve minutes in the Activity box open‐field test, which assesses exploratory behavior. Values are mean ± SEM; n_WT7F_ = 9–10, n_OE7F_ = 6, n_WT15F_ = 12, n_OE15F_ = 10, n_WT22F_ = 7, n_OE22F_ = 8–13

In the 7‐month‐old female PA28αOE mice, we observed improved memory compared to wild type, both in the shuttle box passive avoidance test and in habituation, as well as a reduced depressive‐like behavior using the forced swim test (Figure 5; Adelöf et al., 2018). At 22 months of age, however, PA28αOE and wild type females performed similarly in tests of memory (Figure 5a), habituation (Figure 5b,c), and depressive‐like behavior (Figure 5d). We also found that the reduced depressive‐like behavior of PA28αOE at the 7‐month timepoint was not caused by altered fat mass (Figure 5e), which has been shown to directly influence swimming behavior in the forced swim test (Adelöf et al., 2019). In males, there were no differences between PA28αOE and wild type in memory, habituation, or mobility at 7 months of age (Adelöf et al., 2018), and similar results were seen at the 15 and 22 months' timepoints (Figure S2).

### 2.7 Old female PA28αOE mice display a youth‐like explorative behavior

During mouse aging, exploratory activity is known to markedly decrease (Adelöf et al., 2019; Fahlström et al., 2011). The inclination to explore can be measured by analyzing the mobility of an animal during the first few minutes when introduced to an unfamiliar environment. In the 7‐month‐old female mice, we found no behavioral differences between PA28αOE and wild types during naïve open‐field testing (Figure 5; Adelöf et al., 2018). In the 22 months' cohorts, wild types displayed as expected an “aged” behavior, with horizontal activity reduced by 54% compared to 7‐month‐olds during the first 5 min in the open field test (*p* = 0.0006, Student's *t* test; Figure 5f). In contrast, female PA28αOE 22‐month‐olds were as active as 7‐month‐olds during the first 5 min and thus maintained their exploratory behavior with aging to a markedly higher degree than wild type (*p* = 0.036, Student's *t* test; Figure 5f).

Standing on hind legs—rearing—is considered both exploration and vertical mobility (Tanaka et al., 2012). Age effects on rearing activity during the first 5 min in the open‐field test displayed trends similar to that of exploratory horizontal activity; 22‐month‐old PA28αOE females reared as often as 7‐month‐old wild type and PA28αOE females, which was about twice the frequency of wild types at 22 months of age (Figure 5g).

Exploratory behavior also decreased with aging for males, especially in wild type (Figure S3; horizontal: p_WT7–22_ = 0.0065 Student's *t* test; rearing: p_WT7–22_ = 0.0001, Mann–Whitney). Although there was no difference in exploratory behavior between PA28αOE and wild type males at 22 months of age, PA28αOE males had higher horizontal activity at 15 months (Figure S3a; p_WT15‐OE15_ = 0.023, Student's *t* test), which could indicate maintained youth‐like exploratory behavior also for PA28αOE males but only up until 15 months of age.

## 3 DISCUSSION

We herein demonstrate that the aggregation prevention effect in hippocampus of PA28α overexpressing young adult female mice is maintained as they age. These animals displayed a youth‐like behavior at advanced age; specifically, 22‐month‐olds explored unfamiliar environments to the same extent as 7‐month‐olds. This is indicative of a healthy aging phenotype. As young adults, PA28α overexpressing females had improved learning/memory and reduced depressive‐like behavior. These cognitive effects of PA28α overexpression gradually decreased with age, and there were no differences in these parameters comparing 22‐month‐old wild type and PA28αOE mice. We neither observed any effects on life span or protein damage aging markers. Our results indicate that the elevated capacity to prevent aggregation in hippocampus in PA28αOE females exerts proteostatic effects that cognitively manifests in different ways as the mouse ages: as improved memory in young, and enhanced exploratory behavior in old mice.

**FIGURE 6:**
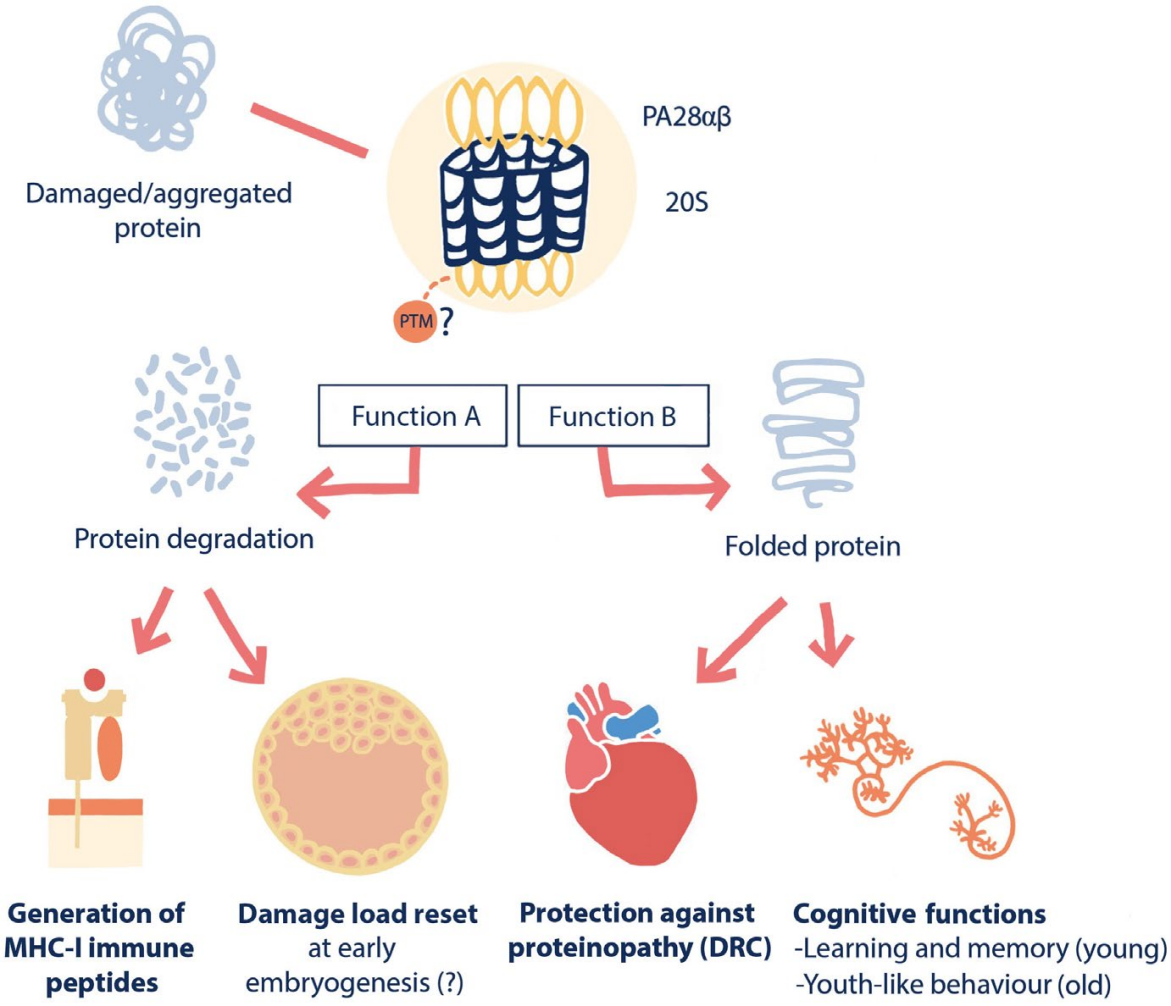
Schematic presentation of PA28αβ's known roles as proteasome activator and as a chaperone. PA28αβ has been linked to diverse physiological functions, including peptide generation in the immune system, protein damage control during early embryogenesis, heart homeostasis (e.g., protection against desmin‐related cardiomyopathy, DRC), and—as presented in this work—cognitive functions. The molecular basis of PA28αβ's role is likely dual and can be separated into its 20S proteasome stimulating (Function A) and chaperone‐like (Function B) activity, possibly orchestrated by PTMs. These two molecular roles seem to act in different physiological functions, as outlined in the figure

The known roles of PA28αβ are schematically summarized in Figure 6; on one hand, PA28αβ exhibits proteasome activation and on the other hand chaperone‐like/anti‐aggregation functions. As laid out below, the molecular shift between these two roles is likely intricately regulated to maintain cellular functions and protein homeostasis though challenges of metabolic stress, inflammation, and aging. PA28αβ as a proteasome activator (function A in Figure 6) is important for generating (at least some) immunopeptides for presentation at the major histocompatibility complex I (MHC‐I; Cascio, 2014; Vigneron & Van den Eynde, 2014), and for protein damage control during early embryogenesis, possibly constituting a proteome rejuvenation check‐point (Hernebring et al., 2006, 2013). PA28αβ as a chaperone, however, (function B in Figure 6), can protect against desmin‐related cardiomyopathy (DRC), a proteinopathy induced by the missense αB‐crystallin mutation CryAB^R120G^ (Li, Horak, et al., 2011). The reasons for why we categorize PA28αβ as exhibiting a chaperone function against DRC is outlined in previous work (Adelöf et al., 2018). Finally, as shown herein, PA28αβ's chaperone‐like functions promote memory and learning at young age and exploration at old age.

That we found no effect of PA28α overexpression on life span or age‐related protein damage was initially unexpected since PA28αβ has been repeatedly associated to the degradation of proteins carrying such deleterious modifications (Hernebring et al., 2013; Pickering & Davies, 2012). Notably, the PA28αβ variant with chaperone‐like function is likely exclusively overrepresented in PA28αOE, and overexpression of the 20S proteasome activating PA28αβ may still affect proteostasis in a manner that could lead to prolonged life span. Such mechanism could theoretically apply to humans as well, since deleterious protein damage (CML) has been shown to dramatically decrease also as human embryonic stem cells (hESCs) differentiate (Barandalla et al., 2017). However, no one has yet to our knowledge investigated PA28‐20S proteasome activity during differentiation of hESCs.

Apart from changes in protein expression, activity of proteasome complexes is known to be regulated by alterations in complex composition and by posttranslational modifications (PTMs) of both 20S core and its activators (reviewed in Kors et al., 2019). As indicated in Figure 6, the function of PA28αβ may be regulated at the protein and/or protein complex level by differences in PTMs of PA28αβ's two functional variants, and this PTM is possibly phosphorylation. An early study reported that phosphorylation of PA28 is required for proteasome activation, as phosphatase treatment abolished PA28‐dependent proteasome activity in rabbit reticulocyte extracts (Li et al., 1996). This discovery has been overlooked since in vitro proteasome capacity studies are commonly performed using recombinant PA28αβ expressed in *E. coli*, which has been supposed unlikely to generate phosphorylated mammalian recombinant proteins (Cascio, 2014). However, since mammalian proteins expressed in E. coli indeed can carry phosphorylations (e.g., Shrestha et al., 2012), this requires further testing. Actually, a considerable number of PTMs of both PA28α and PA28β have been identified in high‐throughput screens, namely phosphorylations, lysine acetylations, methylations, ubiquitinations, and succinylations, as summarized in Phosphosite for PA28α (https://www.phosphosite.org/uniprotAccAction?id=P97371) and PA28β (https://www.phosphosite.org/uniprotAccAction?id=P97372). The functional effects of all these PTMs are unknown. One of them, or several, could potentially constitute a molecular shift behind PA28αβ's two different functions.

In this work, we observed differences between heart and hippocampus in how protein damage levels change upon aging. In the heart, protein damage increased by 25% in protein carbonyl level and 21% in CML, during aging from 7 months (100% survival) to 22 months of age (70%–75% survival), but there was unexpectedly no such effect in hippocampus. A literature search showed that induction of heart protein carbonylation upon aging ranged from 30% to 250% (Gu et al., 2013; Li et al., 2005; Wu et al., 2016), and between 80% and 200% in CML (Li et al., 2005; Ren et al., 2007). Levels of protein carbonyls vary greatly between brain regions in mice. Dubey and colleagues found that protein carbonyls increased with aging of C57BL/6 mice by 30% in hippocampus and 85% in striatum, while there was no difference at all in carbonyl levels with age in hindbrain (Dubey et al., 1996). A 50% increase in protein carbonyls in hippocampus with age in C57BL/6 mice (Choi et al., 2004) and a 200% increase in CML levels in C57BL/6JNia mice (Thangthaeng et al., 2008) have also been reported.

Though reports of unchanged protein damage levels in hippocampus during aging are scarce, our results anyhow indicate an innate difference between how hippocampus and heart age. This notion is supported by a recent study comparing gene expression of age‐related biological pathways in different rat tissues, in which hippocampus showed a distinctive transcriptomic profile. In addition to displaying the smallest fold change of upregulated genes, hippocampus demonstrated unaltered gene expression in pathways associated to mitochondrial function that were the most downregulated by aging in other tissues (Shavlakadze et al., 2019). This may reflect that hippocampus is able to uphold mitochondrial integrity and sustain proteostasis upon aging much more efficiently than other tissues. Such situation would result in a postponed accumulation of protein damage upon aging. Protein carbonyls have been shown to accumulate with age in tissues in a biphasic manner, with an exponential increase in roughly the last third of life span (Levine, 2002). The 22‐month timepoint represents animals with around 1/6 left of their life span, and it is possible that the shift in protein damage accumulation rate occurs even later than this in hippocampus.

Heart and hippocampus also differed in proteasome activity, considering that PA28‐20S activity increased with age in heart (by 67%) but not in hippocampus. Remarkably, the increase in PA28‐20S activity with age in heart occurs without an induction of PA28α levels, strengthening the hypothesis of an unknown molecular regulation behind PA28αβ's dual function as outlined in Figure 6. Proteasome activity is generally considered to decrease with age, though results are conflicting (as reviewed; Koga et al., 2011). However, it is important to note that proteasome activity in cell extracts may be distinct from that in tissue, since aging may induce ATP deficiency and/or protein aggregate interactions that can hamper the proteolytic machinery in vivo and these could be reversed by protein extraction.

We demonstrate herein that female PA28αOE mice exhibit a youth‐like exploratory activity at advanced age. PA28αOE males also maintain elevated exploratory activity up to at least 15 months of age, implying that the effect may exist in males as well although not as strong and prolonged. Exploratory behavior is a basic adaptive response to changing environments and highly important for survival. Definitions of what constitutes exploratory behaviors varies but that mice have an innate attraction to novel stimuli is commonly accepted. Locomotion in a novel environment assessed by open‐field tests allows for robust and translatable measurements of exploration, but notably, there are in general exploratory factors to all behavioral tests as most analyses introduce components previously unknown to the mouse (Adelöf et al., 2019; Walsh & Cummins, 1976).

When encountering a new spatial setting, rodents partly rely on the hippocampus to decipher the novelty of the situation (McDonald et al., 2018) promoting long‐term potentiation which enhances synaptic transmissions and improves learning and memory responses. In addition to stimulate learning, responses to new environments include for example enhanced perception and motivation. In humans, fMRI studies have shown that novel spatial stimuli result in stronger activations across wider ranges of the medial temporal lobe (MTL) as compared to familiar stimuli (Davis et al., 2004). In rodents, differences in exploratory behaviors have been linked to changes in gene expression in several brain regions (such as the prefrontal cortex, amygdala, and periaqueductal gray; Nelovkov et al., 2006) and specific neuromodulatory responses are known to be induced only by novel stimuli (Schomaker & Meeter, 2015). Thus, exploratory activity differs in the neuronal activation as compared to learning and memory, resulting in a distinction of the two behavioral effects of PA28αOE mice from a neuronal response perspective.

The enhanced hippocampal chaperone‐like capacity observed in female but not male PA28αOE mice could explain the cognitive effects in PA28αOE females. Although we do not know the biological cause to this sex discrepancy, it is most likely not directly linked to sex hormonal factors, since we found no differences in the levels of serum β‐estradiol or S105 phosphorylated estradiol receptor β between PA28αOE and wild type females in any of the cohorts. There may be indirect effects, and there are sex‐specific differences in the brain (McCarthy, 2008); female rodents have for example higher density of dendritic spines in hippocampus than males, which changes for the opposite under stressful conditions when males instead obtain greater density of dendritic spines (Shors et al., 2001). However, the increased exploratory behavior of PA28αOE males compared to wild type males at 15 months of age demonstrates that PA28αOE has an effect also in males, although not as pronounced as in females. This effect could in theory also be linked to a chaperone‐like function, albeit present in other brain regions than hippocampus or in a context dependent manner not detected at termination of these animals.

The function of PA28α overexpression in neuronal responses thus needs to be analyzed under different stimuli to be further understood. Still, from this lifespan study of PA28αOE mice, we conclude that overexpression of PA28α results in improved behavior in cognitive tasks at early age and youth‐like behavior at old age, both possibly linked to the chaperone‐like function of PA28αβ.

## 4 EXPERIMENTAL PROCEDURES

### 4.1 PA28αOE mouse model and mouse strain backgrounds

Animals used in the study were C57BL/6N×BALB/c F2 hybrid mice originating from an F1 crossing of C57BL/6N (Charles River) and BALB/c (Harlan Laboratories), except for hippocampi samples from 4‐month‐old mice which were from pure C57BL/6N mice. The PA28α overexpressing mouse model (PA28αOE) was generated previously (as described in Adelöf et al., 2018) through knock‐in technique of PA28α overexpression cassette (CAG promoter to drive the murine coding region of PA28α) at the *Rosa26* locus, and correct integration was verified in ES cells and splenocytes by Targeted Locus Amplification (Cergentis).

### 4.2 Animal care and termination

All mice were housed with a 12:12 h light–dark cycle (dawn: 5.30–6.00, dusk: 17.30–18.00) at room temperature (21°C) with controlled humidity (45%–55%) under daily surveillance. Water and regular chow diet were given *ad lib* (R3; energy percentage: 12% fat, 62% carbohydrates, and 26% protein and a total energy content of 3 kcal/g. Lactamin). Female mice were initially cohoused four by four, but the number decreased as mice died or were euthanized over time in the lifespan experiment. Males were housed in groups of three but changed to single housing at 6 months of age due to aggression and fighting. To maintain representative male cohorts and to not select for dominant males, eleven cages with signs of fighting were removed from the study. Cages were cleaned every week (cohoused animals) or every 2 weeks (single housed animals), and nesting material (paper, cardboard houses, and wooden sticks) was transferred to new cages upon cleaning.

Euthanization took place under anesthesia (5% isoflurane) by decapitation. In the lifespan study, animals were euthanized upon signs of ill‐health (e.g., weak posture, inactivity, shabby fur, failure to eat or drink, enlarged organs and tumors; in line with Adelöf et al., 2019). At termination of mice in the behavioral study cohorts, organs and tissues were collected and directly transferred to dry ice, and kept frozen in −80°C until biochemical analyses. Animal work was carried out in accordance with EU Directive 2010/63/EU for animal experiments, and the study was performed following the ethical certificate approved by the Animal Ethics Committee in Gothenburg, Sweden (Permit Number: 164‐2015).

### 4.3 Study design of lifespan analysis and behavioral phenotypic profiling

The lifespan study was carried out with a total of 200 male and female wild type and PA28αOE mice (n_WTfemales_ = 51, n_OEfemales_ = 60, n_WTmales_ = 48, n_OEmales_ = 41). Cohorts of mice from this lifespan study were subjected to behavioral profiling and organ harvesting at 7, 15, and 22 months of age, when mice were considered mature adults, middle‐age and old, respectively (Adelöf et al., 2019; Flurkey et al., 2007). At the time for phenotypic analyses, the mice were exactly 6.6–7.8 ± 0.2 (age at test period start – age at test period end ±SD of age variance in the cohort; 7 months), 14.5–15.6 ± 0.1 (15 months), 21.8–22.5 ± 0.2 (22 months) and no attempt to control for seasonal effects was made (7 months: September–October; 15 months: May–June; 22 months: late December–January). These cohorts of mice were included in the lifespan analysis and marked as censored at the timepoint of first behavioral assessment. To estimate natural life span, we calculated intervals between two survival curves. The minimum survival curve was obtained by counting euthanized animals as if they had died from natural causes and the maximum survival curve by counting these mice as if they were as healthy as their littermates (as described in Adelöf et al., 2019).

Mice were acclimatized for 1‐week prior initiation of studies, and behavioral analyses known to be affected by handling (such as activity box) were performed early in the testing period (except for females at 15 months in the activity box analysis which therefore was omitted from data analysis) and tests which affect the mice to a greater extent were performed later in order to give the animals longer recovery time and to not impact subsequent analyses. To ensure complete separation of the sexes and no sex‐dependent impact of the analyses, the order of the behavioral test‐battery differed between females and males. Behavioral tests were single‐blind and carried out at daytime (between 10.00 and 14.00) after the mice had been given at least 1 h of acclimatization in the experimental room. Data from the animals from the 7‐month timepoint have previously been published (Adelöf et al., 2018) as well as all wild type mice (Adelöf et al., 2019), and an analysis of cataract incidence of all animals in the lifespan study (Hernebring et al., 2021).

### 4.4 Activity box

General activity, exploratory behavior, and habituation can be measured in the Activity box, an open‐field activity‐like test. In a sound‐proof opaque box (50 × 50 × 50 cm; Kungsbacka mät och regler, Fjärrås, Sweden) with low‐intensity light in the lid, infrared sensors (8L × 8B × 8H) recorded three‐dimensional movements of mice (horizontal activity, peripheral activity, rearing activity, peripheral rearing, rearing time, locomotion, and corner time as beam breaks in 5 min' bins). On the first day, mice were placed in the middle of the box and recorded for 1 h in this novel environment to assess exploratory behavior (first 5 min) and general activity. On the second day, mice were recorded again in the acquainted environment to assess habituation.

### 4.5 Forced swim test

The forced swim test analyses signs of depression, generally referred to as depressive‐like behavior. In transparent plexiglas cylinders (25 cm inner diameter and 60 cm depth), 22°C water was filled in level with a gray, circular platform hanging on the outside of the cylinder 20 cm from the top (bespoke construction, AstraZeneca Gothenburg). The swimming and movement pattern of the mice were recorded by a camera placed on the top of the cylinder for 6 min and 20 s, of which the last 4 min were used for analysis (MouseTracker analysis software).

### 4.6 Shuttle box passive avoidance test

Learning and memory was assessed though passive avoidance analysis in the shuttle box system (Accuscan Instruments Inc.). The shuttle box is a two‐compartment box, one lit (transparent walls) and one dark (opaque walls), connected by a sliding door which can be programmed to open or close, and a cage floor of stainless‐steel grid which can deliver a mild electric shock upon certain stimuli. The passive avoidance test was performed on two consecutive days. On day 1—learning day—mice were placed in the lit compartment and after 30 s the door opened, giving the choice to explore the dark compartment (which mice tend to do). The door closed once the mice had entered the dark compartment and the grid delivered a mild electric shock (0.3 mA). A vocal response and jumping were observed for all mice as a reaction to the discomfort and enabled continuation to day 2—memory day. On day 2, mice were once again placed in the lit compartment and given the choice to enter the dark compartment as the door opened. The time it took for the mice to enter the dark compartment was recorded (maximum 300 s) on both days and no entry or a longer interval on the second day indicated a memory response.

### 4.7 Body composition

Body composition was analyzed by dual‐energy X‐ray absorptiometry (DEXA) using Lunar PIXI‐mus Densitometer (GE Medical Systems) under 2% isoflurane sedation. Parameters analyzed were body fat (g), body length, body fat (%), lean body mass (g), and total BMD (g/cm^2^).

### 4.8 Blood serum preparation and β‐estradiol detection

Blood was collected from the left atrium of the heart under isoflurane anesthesia, prior to necropsy, incubated at room temperature for 30–45 min, centrifuged to isolate serum, which was stored at −80°C until analysis. β‐estradiol levels were detected by the Mouse/Rat Estradiol ELISA‐kit (#ES180S‐100; Calbiotech) according to manufacturer's instructions. The β‐estradiol serum levels are presented as relative values for each separate timepoint, as we found that both kit performance and β‐estradiol in serum at −80°C were not stable over time (months).

### 4.9 Analyses of protein expression and protein damage markers by SDS–PAGE and Western blot

Heart (left ventricle) and left hippocampus were manually grinded in Eppendorf tubes and lysed in a modified RIPA buffer (50 mM Na_2_HPO_4_ pH7.8; 150 mM NaCl; 1% Nonidet P‐40; 0.5% deoxycholate; 0.1% SDS; 1 mM DTPA; 1 mM 4‐(2‐Aminoethyl)benzenesulfonyl fluoride hydrochloride, AEBSF). Debris was removed by centrifugation at 5000 *g* (heart) and 10,000 *g* (hippocampus) for 10 min at 4°C, and protein concentration of the cell extracts was determined using the Pierce™ BCA Protein Assay Kit (Thermo Fisher Scientific). There was no difference in hippocampi protein carbonyl content when comparing centrifugation at either 5000 *g* or 10,000 *g* as demonstrated in Figure S4, confirmed with ImageJ quantification.

Samples were prepared in loading buffer (100 mM Tris‐HCl, 50% Glycerol, 10% sodium dodecyl sulfate (SDS), 0.15% Bromofenol blue, and 0.05% β‐mercaptoethanol), incubated for 5 min in 98°C and loaded onto NuPAGE Novex 4%–12% Bis‐Tris gel for separation by sodium dodecyl sulfate (SDS)‐polyacrylamide gel electrophoresis (PAGE). The proteins were transferred onto nitrocellulose membranes (Invitrogen) with the Hoefer^TM^ TE22 Mighty Small Transfer Tank system. The membranes were probed with rabbit mAb PA28α (1:5000 #9643; Cell Signaling Technology, Inc.), mouse mAb anti‐N^ɛ^Carboxymethyllysine (1:1000 CML26, #125145; Abcam), rabbit mAb anti‐Hsp90 (1:3000 #4877; Cell Signaling Technology, Inc.), rabbit mAb anti‐Hsp40 (1:3000 #4871; Cell Signaling Technology, Inc.), rat mAb anti‐Hsc70 (1:3000 #ab19136; Abcam), and rabbit pAb Estrogen Receptor β phospho S105 (1:3000 #ab62257; Abcam). Carbonylated proteins were detected through 2,4‐Dinitrophenylhydrazine (DNPH) derivatization prior SDS‐PAGE separation, using the OxyBlot^TM^ Oxidized Protein Detection Kit following manufacturer's instructions (Millipore S.A.S). The Odyssey infrared imaging system and software (Odyssey CLx‐0789, LI‐COR Biosciences) was used for detection of secondary antibodies; IRDye 800CW‐labeled goat anti‐rabbit, 800CW‐labeled donkey anti‐goat IgG, 800CW‐labeled goat anti‐rat, and 680CW‐labeled goat anti‐mouse antibodies (LI‐COR Biosciences). Blots were quantified using the ImageJ software. Total protein levels were confirmed using Ponceau membrane staining. There were no differences between the sexes in levels of protein carbonyls and CML (Figure S5), and therefore, the data from male and female tissue were pooled in the statistical analyses.

### 4.10 Proteasome capacity assays

Heart (left ventricle) and hippocampus (right for PA28‐20S and 20S; left for 26S) were manually grinded in Eppendorf tubes in proteasome complex specific lysis buffers: PA28‐20S and 20S (25 mM Tris/HCl pH 8.3, 0.2% Nonidet P‐40) and 26S (25 mM Tris/HCl pH 8.3, 0.2% Nonidet P‐40, 100 mM NaCl, 5 mM ATP pH 7.5, 20% glycerol and, after aliquot taken for BCA, 0.5 mM DTT). Samples were thereafter centrifuged at 5000 *g* for 10 min at 4°C to remove cell debris, and protein concentration was determined with BCA Protein Assay kit (Pierce, Thermo Fisher Scientific). PA28‐20S, 26S, and 20S proteasome chymotryptic activity was analyzed though hydrolysis of the fluorogenic peptide succinyl‐Leu‐Leu‐Val‐Tyr‐7‐amino‐4‐methylcoumarin (suc‐LLVY‐AMC; BostonBiochem, Bio‐Techne) as described (Hernebring, 2016) with some modifications. Samples of 20 µg (heart) and 10 µg (hippocampus) total protein were incubated with 200 µM suc‐LLVY‐AMC in proteasome complex specific assay buffers: PA28‐20S (50 mM Tris/HCl pH 8.3, 0.5 mM DTT), 20S (50 mM Tris/HCl pH 8.3, 0.5 mM DTT, and 0.02% SDS) and 26S (50 mM Tris/HCl pH 8.3, 5 mM ATP, 0.5 mM DTT) in a total volume of 100 µl. Fluorescence was monitored using 390 nm excitation and 460 nm emission filters. Free AMC was used as standard control (Molekula Ltd.), and activity was determined as the slope of fluorescence over time (120 min) and calculated per ug protein. Protein levels in assays were determined by SDS–PAGE followed by Coomassie staining of the gel (0.1% Coomassie Brilliant Blue R‐250, 40% Methanol, 10% acetic acid) and using the Odyssey infrared system and software for imaging (LI‐COR Biosciences). Values are given as total suc‐LLVY hydrolysis capacity but to control for proteasome independent/background activity samples were treated with 5 µM epoxomicin proteasome inhibitor (Sigma‐Aldrich). Epoxomicin inhibition of proteasome capacity (mean ± SD) in wild type was for heart: PA28‐20S 77.1 ± 5.2%, 26S 95.6 ± 1.7%, 20S 88.9 ± 4.6%; and hippocampus: PA28‐20S 75.8 ± 5.1%, 26S 90.6 ± 2.7%, 20S 96.3 ± 3.3%. There were no differences between the sexes in PA28‐20S, 20S, and 26S proteasome capacity (Figure S6), and data from male and female tissues were therefore pooled in the statistical analyses.

### 4.11 Luciferase aggregation prevention

Hippocampus were analyzed for luciferase aggregation prevention capacity as previously described (Adelöf et al., 2018). Specifically, right hippocampi were manually grinded in Eppendorf tubes and lysed in extraction buffer (25 mM Tris/HCl pH 7.8, 100 mM NaCl, 5 mM MgCl_2_, 1 mM ATP, 1 mM AEBSF, and 5% glycerol), and cell debris was removed by centrifugation at 5000 *g* for 10 min followed by addition of 1 mM DTT after an aliquot was taken for protein concentration determination with the BCA Protein Assay kit (Pierce, Thermo Fisher Scientific). Luciferase (200 nM; SRE0045, Sigma‐Aldrich) was denatured at 42°C in 50 mM Tris (pH 7.6) and 2 mM EDTA, in the presence of 5 µg hippocampal protein extracts (4.5 µg for females 7 months) and aggregation of luciferase was measured as light scattering at 340 nm. Sample wells without protein extract were used as positive control (reference indicating maximum aggregation). Protein samples without luciferase were used as negative control of aggregation and extraction buffer only was considered background, neither of these controls changed over time. The chosen timepoint for analysis was when the positive control (without protein extract) had reached 60%–80% of maximum aggregation. The luciferase aggregation prevention capacity of the hippocampus extracts was calculated as percent non‐aggregated luciferase.

### 4.12 Statistical analysis

All groups were analyzed for normal distribution by Shapiro–Wilk and QQ plots and homogeneity of variance by Levene's test or homoscedasticity plots. Two‐tailed independent *t* test (Student's *t* test) was used for groups which passed test of normality and equal variance, two‐tailed independent unequal variance *t* test (Welch's test) for normal distributed groups that did not have equal variance, and nonparametric Mann–Whitney test for unevenly distributed groups. Differences between wild type and PA28αOE at the three different timepoints, age effects, and time × group interactions were calculated with 2‐way ANOVA, followed by Sidak multiple comparisons test, for groups with equal *n* and Mixed‐model effect for groups with missing values. Statistics were calculated using GraphPad Prism 8 and IBM SPSS Statistics 27.

## CONFLICT OF INTEREST

The authors declare that they have no conflict of interest.

## AUTHOR CONTRIBUTIONS

JA, JW, MZ, and MH designed research; JA and MH performed research; JA, JW, MZ, and MH analyzed and interpreted the data; JA and MH wrote the manuscript; all authors critically revised the manuscript for important intellectual content and approved the final manuscript.

## Supporting information

Supplementary Material

Supplementary Material

## Data Availability

The data that support the findings of this study are available from the corresponding authors upon reasonable request.
