# Supplementary material for: PA28α overexpressing female mice maintain exploratory behavior and capacity to prevent protein aggregation in hippocampus as they age
Source: Aging Cell. 2021 Mar 15;20(4):e13336. doi: 10.1111/acel.13336 (PMC8045925; doi:10.1111/acel.13336)
Supplement: Supplementary file 1 — Supplementary Material [file ACEL-20-e13336-s001.pdf]

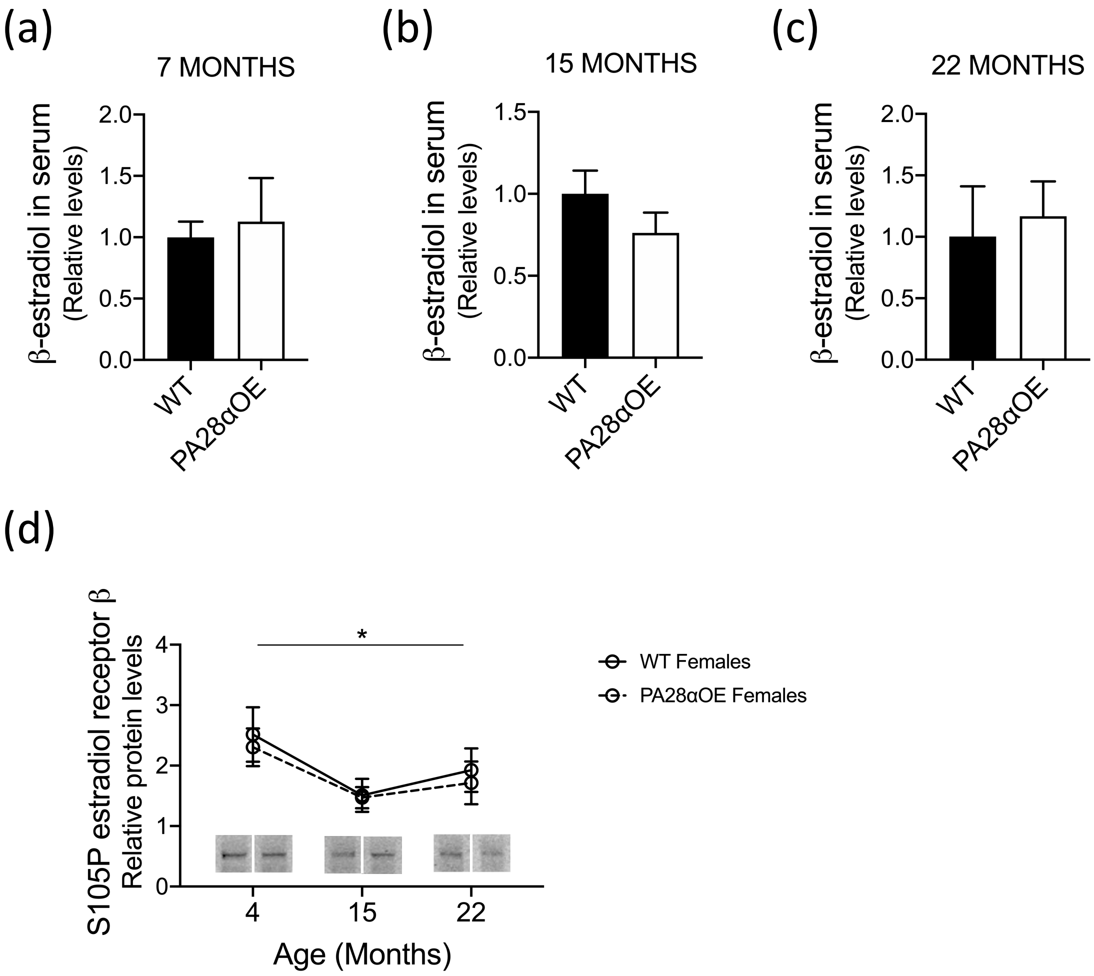

FIGURE S1. Serum β-estradiol levels in PA28αOE and wildtype (WT) females at (a) 7, (b) 15 and (c) 22 months of age (all from F2 hybrid lifespan analysis). Protein levels of (d) S105-phosphorylated estradiol receptor β (all from F2 hybrid lifespan analysis except 4-month-olds which were C57BL/6N). Insets are representative western blots of the different ages (cropped from the same membrane for each age and WT to PA28αOE comparison; WT to the left and PA28αOE to the right). Values are mean ± SEM; Serum estradiol: n=5-11; S105P estradiol receptor β: n=4.

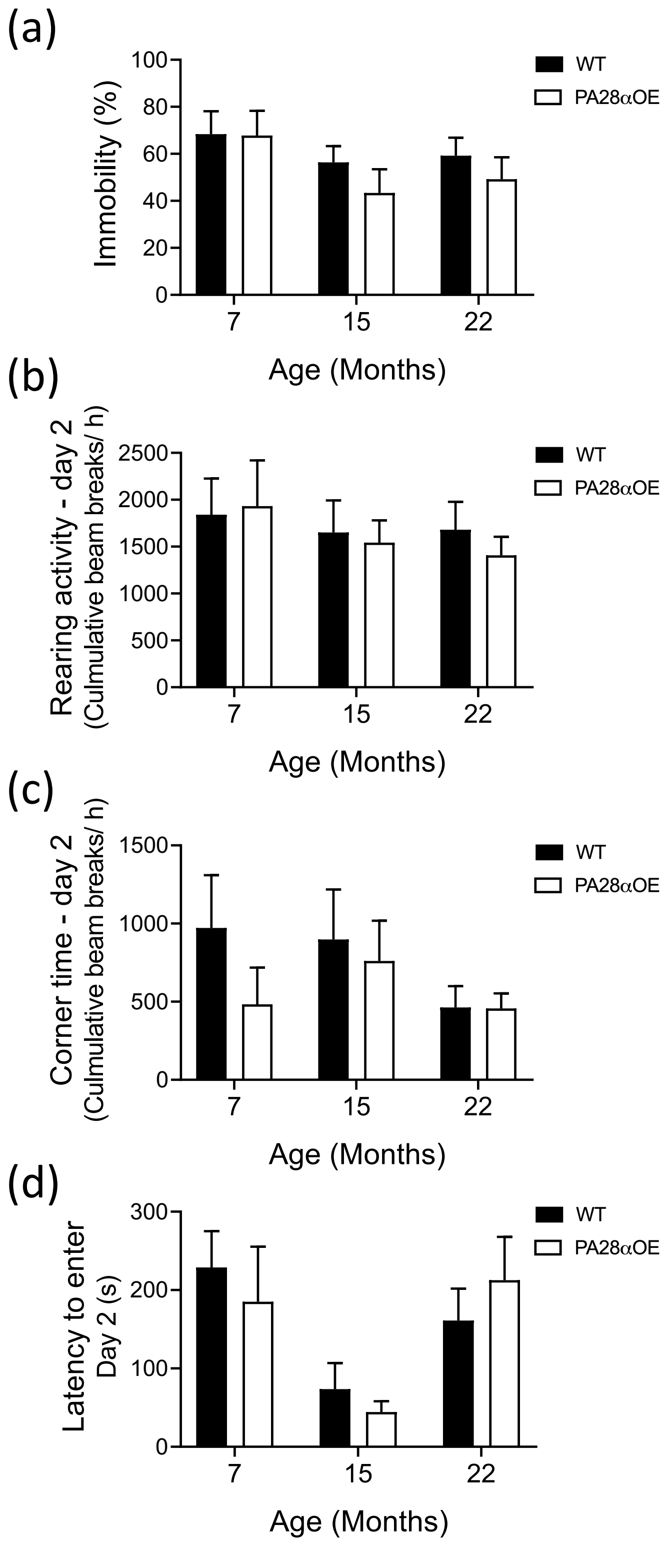

FIGURE S2. Behavioral assessment of wildtype (WT) and PA28αOE males in the F2 hybrid lifespan analysis. In contrast to females, PA28α overexpressing males did not at any time point differ from wildtype males in (a) learning and memory assessment by Shuttle-box passive avoidance test, habituation as analyzed with (b) rearing activity and (c) time spent in corners of an acquainted environment, or (d) immobility in the forced swim test, which is a measurement of depressive-like behavior. Values are mean ± SEM; n_{WT7}=7, n_{OE7}=4-5, n_{WT15}=10, n_{OE15}=10, n_{WT22}=10, n_{OE22}=6-9.

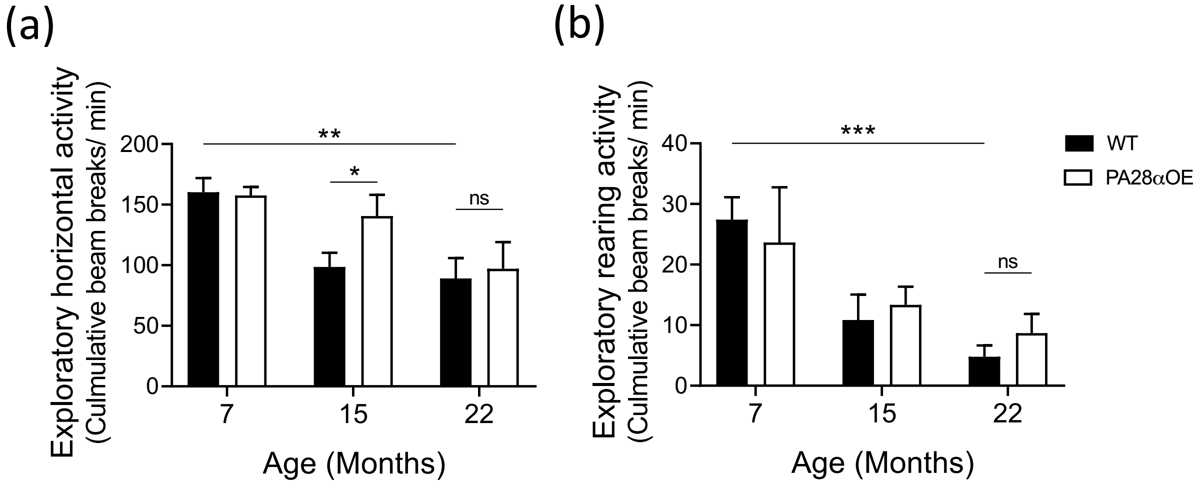

FIGURE S3. Exploratory activity of PA28αOE male mice at 7, 15 and 22 months of age in the F2 hybrid lifespan analysis. Exploratory behavior can be measured during the first 5 minutes in open field test, such as the Activity box. Decrease in exploratory activity is a behavioral marker of aging. (a) Wildtype (WT) mice demonstrate a decline in horizontal activity from 7 to 22 months of age (p_{WT7-22}=0.0065; Student's t-test) which was not observed for PA28αOE males. At 15 months PA28αOE male mice had maintained their activity which was significantly higher than that of wildtype ($p_{WT15-OE15}$=0.023; Student's t-test), this difference was not observed at 22 months of age. (b) Rearing activity declines with age for WT mice (p_{WT7-22}=0.0001; Mann-Whitney) but no statistical significance was found for PA28αOE male mice. Values are mean ± SEM; n_{WT7}=7, n_{OE7}=4, n_{WT15}=10, n_{OE15}=10, n_{WT22}=10, n_{OE22}=9.

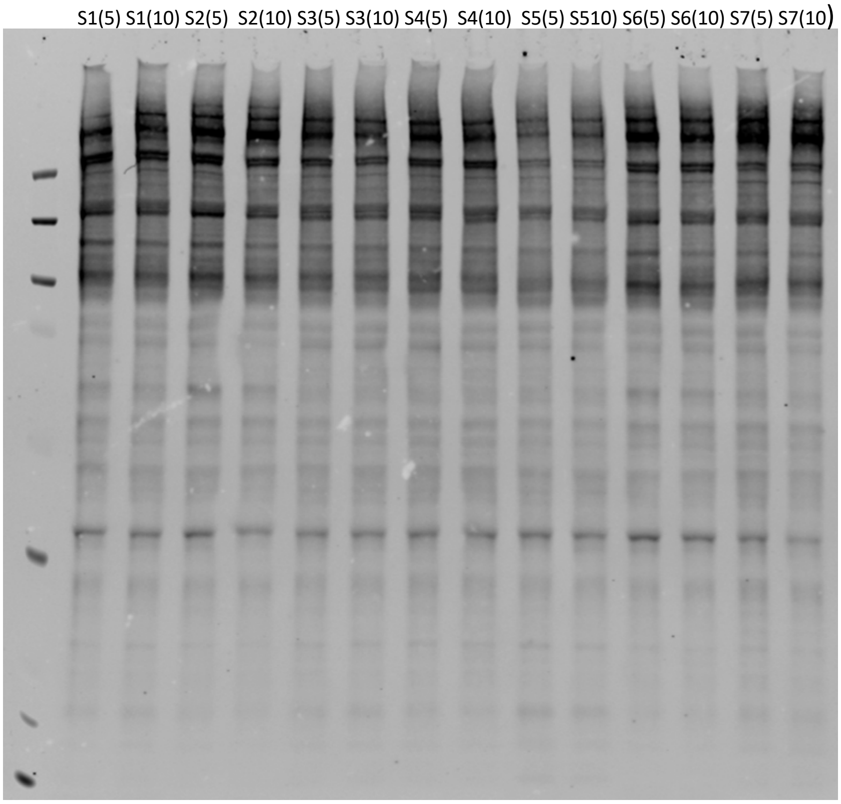

FIGURE S4. Comparison of carbonylated proteins for extracts centrifuged at 5000g or 10000g (RCF). There is no difference in levels of carbonylated proteins by DNPH derivatization if 5000 or 10000 g (RCF) is used as centrifugation for removal of cell debris.

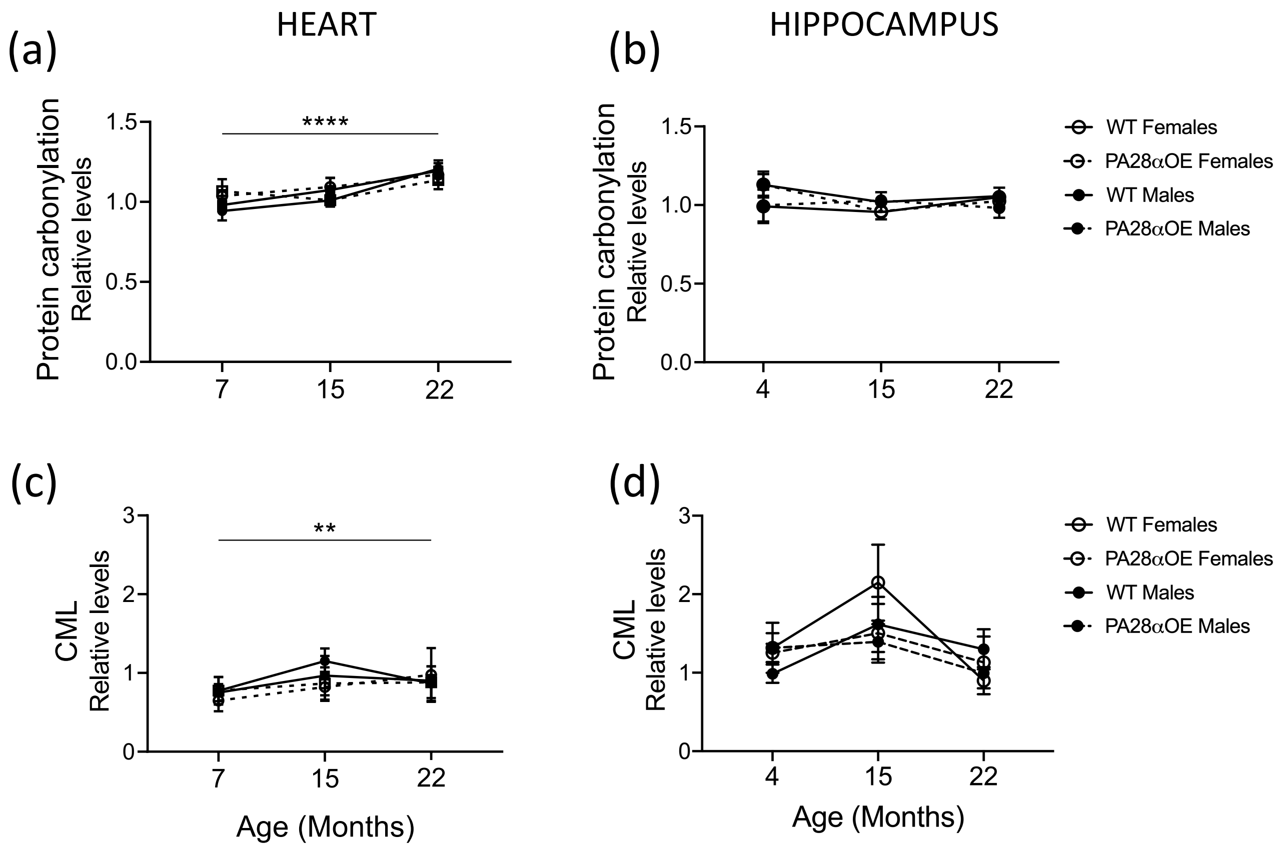

FIGURE S5. Protein damage levels in heart (left) and hippocampus (right) of PA28αOE and wildtype female and male mice. Protein carbonylation in (a) heart and (b) hippocampus. N^{ε}-carboxymethyllysine (CML) in (c) heart and (d) hippocampus. No sex effect was found for PA28αOE or wildtype (WT) mice in levels of carbonylated proteins or CML in heart or hippocampus. Protein carbonylation and CML levels increase with age in heart ($p_{7-22}<0.0001$ and $p_{7-22}=0.0011$, respectively; Mixed-effect model). All tissues are from F2 hybrid lifespan analysis except 4-months hippocampi that are from C57BL/6N. Values are mean ± SEM; heart: n=5-6, hippocampus: n=3-4.

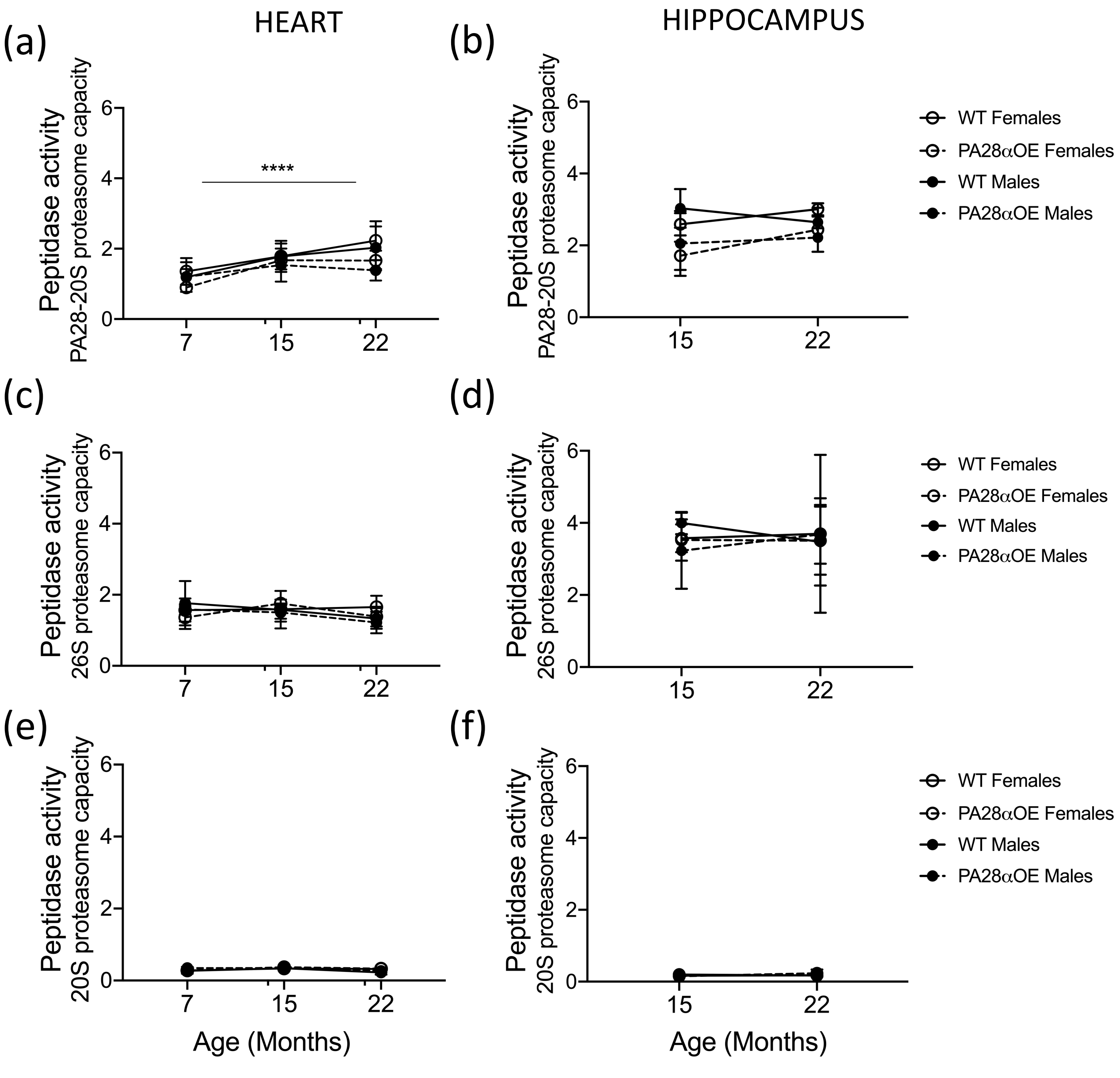

FIGURE S6. PA28-20S, 26S, and 20S proteasome capacity in heart (left) and hippocampus (right) from PA28αOE and wildtype (WT) female and male mice. PA28-20S proteasome capacity in (a) heart ($p_{7\text{-}22}$<0.0001; Mixed-effects model) and (b) hippocampus. 26S proteasome capacity in (c) heart and (d) hippocampus. 20S proteasome capacity in (e) heart and (f) hippocampus. There are no sex differences in PA28αOE or wildtype mice for either proteasome capacity ($p_{WTF\text{-}WTM}$=ns and $p_{OEF\text{-}OEM}$=ns, Mixed-effects model and 2-way ANOVA). All tissues are from the F2 hybrid lifespan analysis. Heart: n=6 except for n_{OEM7}=5, hippocampus: n=3 except for $n_{PA28\text{-}20S(WTF22)}$=2, $n_{26S(WTF22)}$=2, $n_{26S(WTM15)}$=2, $n_{26S(OEM15)}$=4, $n_{20S(WTF22)}$=2 and $n_{20S(OEM15)}$=2. Values are mean ± SD.
