# Supplementary material for: PA28α overexpressing female mice maintain exploratory behavior and capacity to prevent protein aggregation in hippocampus as they age
Source: Aging Cell. 2021 Mar 15;20(4):e13336. doi: 10.1111/acel.13336 (PMC8045925; doi:10.1111/acel.13336)
Supplement: Supplementary file 2 — Supplementary Material [file ACEL-20-e13336-s002.docx]

**TABLE S1.** Physiological parameters of wildtype and PA28αOE F2 hybrid mice.

|  |  |  | BL | BW | Body Temp | BMD | BMC | Fat mass | Lean mass |  |  |  |  |
| --- | --- | --- | --- | --- | --- | --- | --- | --- | --- | --- | --- | --- | --- |
|  |  |  | (cm) | (g) | (ºC) | (mg/cm^2^) | (g) | (g) | (g) |  |  |  |  |
| MALES | 7 | WT | 10.8 ± 0.11 | 39.5 ± 2.2 | 36.7 ± 0.18 | 56.5 ± 1.34 | 0.62 ± 0.03 | 9.4 ± 1.2 | 25.5 ± 0.8 |  |  |  |  |
|  |  | **OE** | **10.6 ± 0.11** | **35.4 ± 1.8** | **36.8 ± 0.12** | **55.8 ± 1.07** | **0.58 ± 0.02** | **7.5 ± 1.3** | **23.7 ± 0.6** |  |  |  |  |
|  | 15 | WT | 10.7 ± 0.11 | 39.9 ± 2.1 | 37.2 ± 0.11 | 57.1 ± 1.05 | 0.65 ± 0.02 | 9.9 ± 1.2 | 25.4 ± 1.0 |  |  |  |  |
|  |  | **OE** | **10.8 ± 0.12** | **44.0 ± 2.0** | **36.9 ± 0.14** | **56.9 ± 1.02** | **0.60 ± 0.02** | **11.5 ± 1.2** | **27.3 ± 0.9** |  |  |  |  |
|  | 22 | WT | 10.8 ± 0.11 | 39.9 ± 1.9 | 36.5 ± 0.13 | 56.3 ± 1.13 | 0.65 ± 0.02 | 9.9 ± 1.7 | 28.7 ± 0.5 |  |  |  |  |
|  |  | **OE** | **11.0 ± 0.14** | **39.4 ± 1.4** | **36.2 ± 0.13** | **54.2 ± 1.32** | **0.60 ± 0.02** | **8.0 ± 1.1** | **29.2 ± 1.3** |  |  |  |  |
| FEMALES | 7 | WT | 10.3 ± 0.09 | 34.6 ± 1.6 | 38.4 ± 0.16 | 56.8 ± 0.84 | 0.56 ± 0.02 | 11.0 ± 0.8 | 20.0 ± 0.6 |  |  |  |  |
|  |  | **OE** | **10.2 ± 0.07** | **30.2 ± 1.6** | **38.3 ± 0.24** | **55.3 ± 1.17** | **0.56 ± 0.02** | **8.68 ± 1.2** | **18.5 ± 0.5** |  |  |  |  |
|  | 15 | WT | 10.5 ± 0.11 | 39.3 ± 2.6 | 37.6 ± 0.10 | 49.3 ± 4.58 | 0.51 ± 0.04 | 14.1 ± 1.8 | 20.7 ± 0.9 |  |  |  |  |
|  |  | **OE** | **10.6 ± 0.09** | **41.1 ± 2.3** | **37.2 ± 0.18** | **57.2 ± 0.93** | **0.58 ± 0.02** | **18.7 ± 3.0** | **20.5 ± 0.6** |  |  |  |  |
|  | 22 | WT | 11.0 ± 0.12 | 36.1 ± 1.9 | 37.7 ± 0.18 | 56.8 ± 1.35 | 0.63 ± 0.04 | 10.6 ± 1.6 | 23.5 ± 1.0 |  |  |  |  |
|  |  | **OE** | **11.0 ± 0.08** | **38.6 ± 2.0** | **37.8 ± 0.16** | **60,3 ± 1.06** | **0.68 ± 0.03** | **14.1 ± 1.7** | **22.2 ± 0.7** |  |  |  |  |

Body composition and core temperatures were analyzed by dual energy X-ray absorptiometry (DEXA) and rectal probe thermometer. No statistically significant differences were found between wildtype and PA28αOE males or wildtype and PA28αOE females. WT: n_M7_=7, n_M15_=10, n_M22_=10, n_F7_=9, n_F15_=12, n_F22_=7; PA28αOE: n_M7_=5, n_M15_=10, n_M22_=9, n_F7_=6, n_F15_=12, n_F22_=12. Mean ± SEM.
